# Hygiène

**Published:** 1838-01

**Authors:** 


### HYGIÈNE.

### *Diseases of Cutlers.* By A. CHEVALLIER.

As M. Chevallier continues to publish monographs on the diseases of artisans, we shall persevere in giving a condensed notice of them. The present article, although it necessarily contains less important matter than the previous ones, on the diseases of printers and lead manufacturers, yet is compiled with similar pains-taking and industry. The grinders of cutlery are exposed to accidents from the breaking of their grinding stones, which may be owing, 1, to the centrifugal force; 2, to the dilatation of the wooden wedges by which the iron axle-tree is fixed into the stones; 3, to the mill-stone being badly mounted, so that it is liable to be dismounted and break. The workmen may also wound themselves with the sharp instruments they are grinding, and when the mills are put in motion by mechanical means the apparatus may injure them. M. Chevallier proves the truth of these statements by cases from various treatises on medical police. The information which he has himself collected was furnished by M. G. Chevallier, a large manufacturer, and by his own observations in a journey to Nogent, (Haute-Marne,) in the neighbourhood of which place 3,300 men are employed in the manufactory of cutlery. In this district ten severe accidents have occurred from the breaking of millstones; the injuries were chiefly confined to the head and face, wounding the cheeks, jaws, and lips, and breaking the teeth. At the Royal Manufactory of Arms at Klingenthal, where sabres are sharpened and cuirasses polished, seven or eight large mills have broken in five years and two workmen been injured. The wounds which the men sometimes receive when sharpening instruments are owing either to awkwardness, and this is very rare, or to grease on the stone causing the instrument to slip rapidly over the mill-stone, and thus to deceive the workman. Two accidents took place during twenty years at Nogent, from the workmen's clothes becoming entangled in the machinery. The precautions which are taken to avoid accidents are the following. The mill-stones are carefully examined before they are purchased, and if there are cracks they are rejected. Some workmen try the stones, by turning them very rapidly before they use them; at the manufactory of arms at Klingenthal this precaution is always taken, the stones being revolved as rapidly as possible for three or four hours; during the last five years four or five mill-stones broke during this trial. In some of the larger workshops the grinding stones are guarded by a semicircular hoop of iron fixed by its two extremities to the stone trough, and an iron bar fixed at one end to the centre of this hoop, and at the other to the end of the trough. Should the stone break, this apparatus is calculated to prevent the fragments from striking the head and face of the workmen, which parts are most frequently injured in these accidents. Instead also of fixing the iron axle-tree into the stone by means of wooden wedges, they are fastened by a nut and screw guarded by leather soaked in oil.

The workmen are subject to varicose veins, and ulcers of the legs; the mill when used dry is dangerous to the grinders, and badly ventilated and imperfectly lighted workshops warmed with coal are injurious to the health. The sight of some is weakened by not using lamps throwing the light from above, and shades, so that they are obliged to give up their trade at an age when other workmen who have taken precautions can still follow it. Apprentices when placed too young at the vice ("étau") often become deformed. The workmen in the neighbourhood of Nogent (amounting to 3,300) are more sober than they were thirty or forty years since, and it does not appear that their trade shortens their lives. They often die from imprudence, and from carelessness regarding the preservation of their health, but they appear to live to an old age, for those who will not take precautions with their eyesight, are sometimes obliged to *relinquish* their trade (which is a profitable one,) at fifty-five or sixty years of age.

*Advice to Master Cutlers.* In order to preserve the health of their men they should, 1, have their mills mounted and guarded so as to prevent accidents; 2, prevent grease from soiling the mills; 3, surround those parts of the machinery which might injure the men, with gratings of wood, or iron, or wire; 4, guard against great rapidity of motion in the mills; 5, examine the mill-stones previously to mounting them; 6, prevent the apprentices from placing themselves in such positions as produce deformity; 7, ventilate the shops, and place them in dry situations.

*Advice to the Workmen.* 1. To use sky-light lamps, shades, and even preservative glasses in time to prevent weakness of sight. 2. To wear laced stockings if they have varices. 3. To live soberly. 4. To guard against placing themselves in bad positions. 5. To avoid exposing themselves to cold when hot, and to wear wooden shoes when they work in low and moist places. 6. To use moderate exercise on their days of rest, instead of frequenting beer-shops (cabarets). 7. To economize, and place the savings in saving-banks. 8. To establish benefit clubs as a provision for illness and old age. It is much to be desired that boys should not be apprenticed at too early an age before they are sufficiently strong to bear the fatigue, otherwise it enervates them, and makes them prematurely old.

*Annales d'Hygiène Publique et de Médecine Légale. Avril,* 1836

### MEDICAL STATISTICS.

